# Intensive lipid-lowering therapy-related regression of a vulnerable plaque confirmed by serial optical coherence tomography: a case report

**DOI:** 10.3389/fcvm.2026.1696537

**Published:** 2026-02-26

**Authors:** Junru Qu, Kun Liu, Neil Johnson, Kenji Wagatsuma, Yingying Su, Beibei Du, Yuquan He

**Affiliations:** 1 Department of Cardiology, China-Japan Union Hospital of Jilin University, Jilin Provincial Cardiovascular Research Institute, Changchun, China; 2 Tsukuba Heart Center, Tsukuba Memorial Hospital, Ibaraki, Japan; 3 Changchun University of Chinese Medicine, Changchun, China

**Keywords:** acute coronary syndrome, case report, inclisiran, lipid-lowering therapy, vulnerable plaque

## Abstract

**Introduction:**

Acute coronary syndrome (ACS) remains a leading cause of mortality and disability worldwide. Vulnerable plaques constitute a key pathological substrate underlying ACS. Although lipid-lowering therapy (LLT) is central to plaque stabilization and secondary prevention in ACS, a substantial proportion of patients fail to achieve recommended lipid targets, underscoring a persistent gap between guideline recommendations and real-world outcomes. Serial assessment of plaque morphology using high-resolution intracoronary imaging may provide clinically actionable guidance for individualized management. Here, we report a case in which a ruptured coronary plaque in a patient with acute myocardial infarction underwent progressive healing, remaining phenotypically vulnerable at early follow-up before ultimately evolving into a stable plaque under intensive lipid-lowering and dual antiplatelet therapy, as documented by serial optical coherence tomography (OCT).

**Case presentation:**

A 52-year-old patient presented with acute chest pain and was diagnosed with ST-segment elevation myocardial infarction. Coronary angiography (CAG) and thrombus aspiration restored coronary flow, after which OCT evaluation was performed. A deferred stenting strategy was adopted, combined with intensive lipid-lowering therapy and dual antiplatelet therapy. At the 1-month follow-up, repeat CAG and OCT supported continued conservative management, and stent implantation was not performed. Over the subsequent year, the patient achieved sustained lipid control under close follow-up, without recurrent chest pain or other cardiac-related symptoms. Conservative management was continued without stent implantation. Serial OCT at 1 month and 1 year demonstrated a stepwise morphological transition of the culprit lesion: from an initially ruptured plaque to a healed but still vulnerable phenotype at early follow-up, and ultimately to a stable plaque phenotype at 1 year.

**Conclusion:**

Vulnerable plaques represent a principal pathological driver of acute coronary events. Sustained and effective LLT promotes plaque stabilization and regression, while serial OCT, owing to its high resolution and reproducibility, enables dynamic assessment of plaque morphology and supports individualized management in patients with ACS.

## 1 Introduction

Acute coronary syndrome (ACS) is a leading cause of mortality and disability worldwide, with substantial risk extending beyond the acute phase to long-term cardiovascular events (1). Vulnerable plaques are a key pathological substrate of ACS, serving as both a trigger for acute coronary events and a marker of future cardiovascular risk (2, 3). Despite the established role of lipid-lowering therapy (LLT) in plaque stabilization and secondary prevention, a substantial proportion of patients with ACS fail to achieve recommended lipid targets in clinical practice (4). Accordingly, more intensive and individualized lipid-lowering strategies—including therapies targeting proprotein convertase subtilisin/kexin type 9 (PCSK9)—have been incorporated into contemporary management of high-risk patients (5, 6). In parallel, serial optical coherence tomography (OCT)—with an axial resolution of approximately 10–15 μm and good reproducibility—allows longitudinal assessment of plaque morphology and healing (3, 7). In this case, we report a patient with ST-segment elevation myocardial infarction (STEMI) in whom intensive lipid-lowering therapy was associated with progressive plaque healing. In particular, serial coronary angiography (CAG) and OCT at 1 month and 1 year demonstrated thrombus resolution and morphological stabilization of the initially ruptured plaque.

## 2 Case presentation

**Figure 1:**
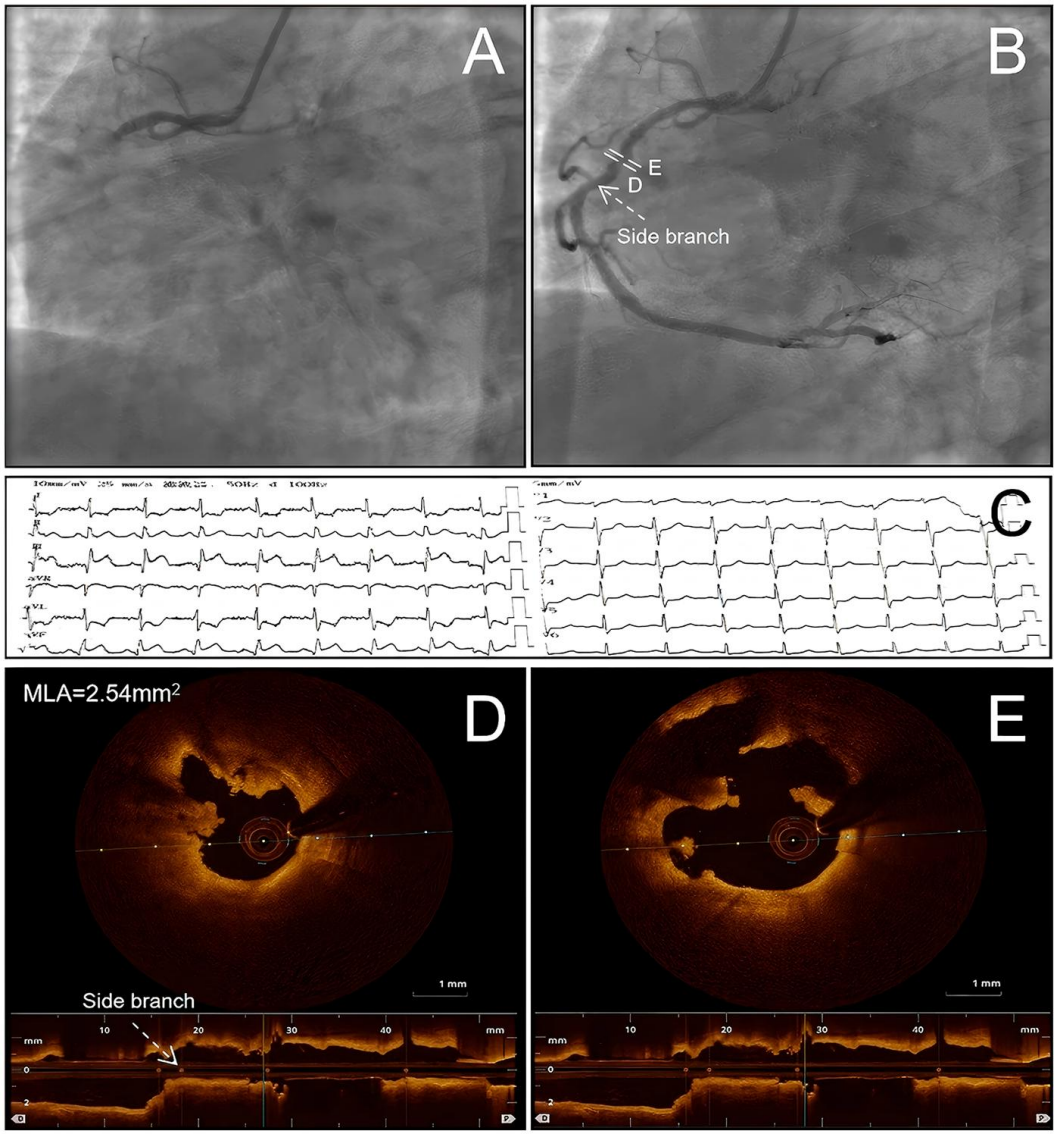
Admission electrocardiogram (ECG), initial CAG and OCT. Initial coronary angiography (CAG) showed total occlusion of the proximal right coronary artery (RCA) **(A)**. Repeat CAG after intracoronary thrombus aspiration (TA) demonstrated moderate residual stenosis in the proximal RCA **(B)**. The admission ECG showed ST-segment elevation in the inferior leads **(C)**. Baseline OCT revealed a minimal lumen area (MLA) of 2.54 mm^2^ **(D)** and demonstrated fibrous cap disruption with a false lumen at the site of plaque rupture **(E)**. CAG, coronary angiography; ECG, electrocardiogram; MLA, minimal lumen area; OCT, optical coherence tomography; RCA, right coronary artery; TA, intracoronary thrombus aspiration.

A 52-year-old man was admitted to the emergency department on 11 March 2024, with persistent chest pain lasting for 3 h, which was not relieved despite repeated sublingual administration of nitroglycerin (a cumulative dose of 5.0 mg, 0.5 mg per tablet, 10 tablets in total). He had no prior history of hypertension or diabetes mellitus and reported occasional smoking. There was no known history of coronary artery disease or long-term use of cardiovascular medications before admission. His father had a history of hyperlipidemia and cardiovascular disease. Physical examination revealed no significant abnormalities. Emergency electrocardiography (ECG) showed ST-segment elevation in the inferior leads (Figure 1C). Acute inferior wall STEMI was diagnosed.

The patient received aspirin (300 mg) and ticagrelor (180 mg). Emergency CAG revealed total occlusion of the proximal right coronary artery (RCA) with thrombolysis in myocardial infarction (TIMI) flow grade 0 (Figure 1A). The left coronary system demonstrated only mild, non-obstructive stenosis in the mid left anterior descending (LAD) artery with preserved TIMI 3 flow, without other angiographically significant stenoses. A 0.014-in Runthrough guidewire (Terumo Corporation, Tokyo, Japan) crossed the occluded segment of the proximal RCA. Intracoronary thrombus aspiration (TA) was then performed, followed by intracoronary administration of tirofiban (0.5 mg). After thrombus aspiration, coronary flow was restored to TIMI flow grade 3, and the patient's chest pain was relieved. Repeat CAG demonstrated moderate residual stenosis in the proximal RCA with TIMI flow grade 3 (Figure 1B). This was initially assessed visually by experienced operators and subsequently confirmed offline using semiautomated quantitative coronary angiography (Anythink cardiovascular analysis software, CREALIFE, Beijing, China) performed by an experienced analyst. Subsequently, OCT was performed to achieve high-resolution intravascular characterization of the culprit lesion. OCT delineated the underlying mechanism of the acute coronary event, evaluated the plaque–thrombus interface following thrombus aspiration, and established a high-quality morphological baseline for subsequent risk stratification, clinical decision-making, and imaging-based follow-up. OCT demonstrated extensive homogeneous, low-attenuation signals with minimal shadowing, consistent with platelet-rich white thrombus. Plaque rupture was also identified, characterized by a discontinuous fibrous cap and formation of a false lumen (Figure 1E and Supplementary Video S1). The minimal lumen area (MLA) of the culprit lesion was 2.54 mm^2^ (Figure 1D). At that time, the patient was hemodynamically stable and free of chest pain. After shared decision-making, the patient and his family declined stent implantation. Given restored TIMI 3 flow after thrombus aspiration and the absence of recurrent ischemic symptoms, a deferred stenting strategy with guideline-directed medical therapy (GDMT) and close follow-up was adopted. Accordingly, intensive lipid-lowering therapy and dual antiplatelet therapy were initiated as part of the deferred stenting strategy.

Laboratory testing demonstrated elevated cardiac injury biomarkers: troponin I at 1.03 ng/mL (reference range, 0–0.05 ng/mL), creatine kinase-MB at 6.38 ng/mL (reference range, 0–3.38 ng/mL), and myoglobin at 38.1 ng/mL (reference range, 0–107 ng/mL). Lipid profiling revealed triglycerides of 2.29 mmol/L, total cholesterol of 6.69 mmol/L, and low-density lipoprotein cholesterol (LDL-C) of 4.13 mmol/L. Other laboratory parameters were within normal limits. Transthoracic echocardiography demonstrated a left ventricular ejection fraction of 58%, and chest radiography revealed no obvious abnormalities.

Lifestyle modifications were recommended, including smoking cessation and adherence to a low-salt, low-fat diet. Tirofiban was administered for 24 h after the procedure to prevent acute thrombotic reformation. Long-term oral medications included aspirin (100 mg once daily) and ticagrelor (90 mg twice daily) for dual antiplatelet therapy, metoprolol (47.5 mg once daily) for secondary prevention, and atorvastatin (20 mg once daily) for lipid lowering. In addition, to intensify lipid-lowering therapy, the patient was advised to receive a scheduled course of subcutaneous inclisiran (284 mg), with injections administered at baseline, 3 months, and 9 months. The patient experienced no recurrent chest pain during hospitalization and was discharged on hospital day 7 with the aforementioned oral medications.

**Figure 2:**
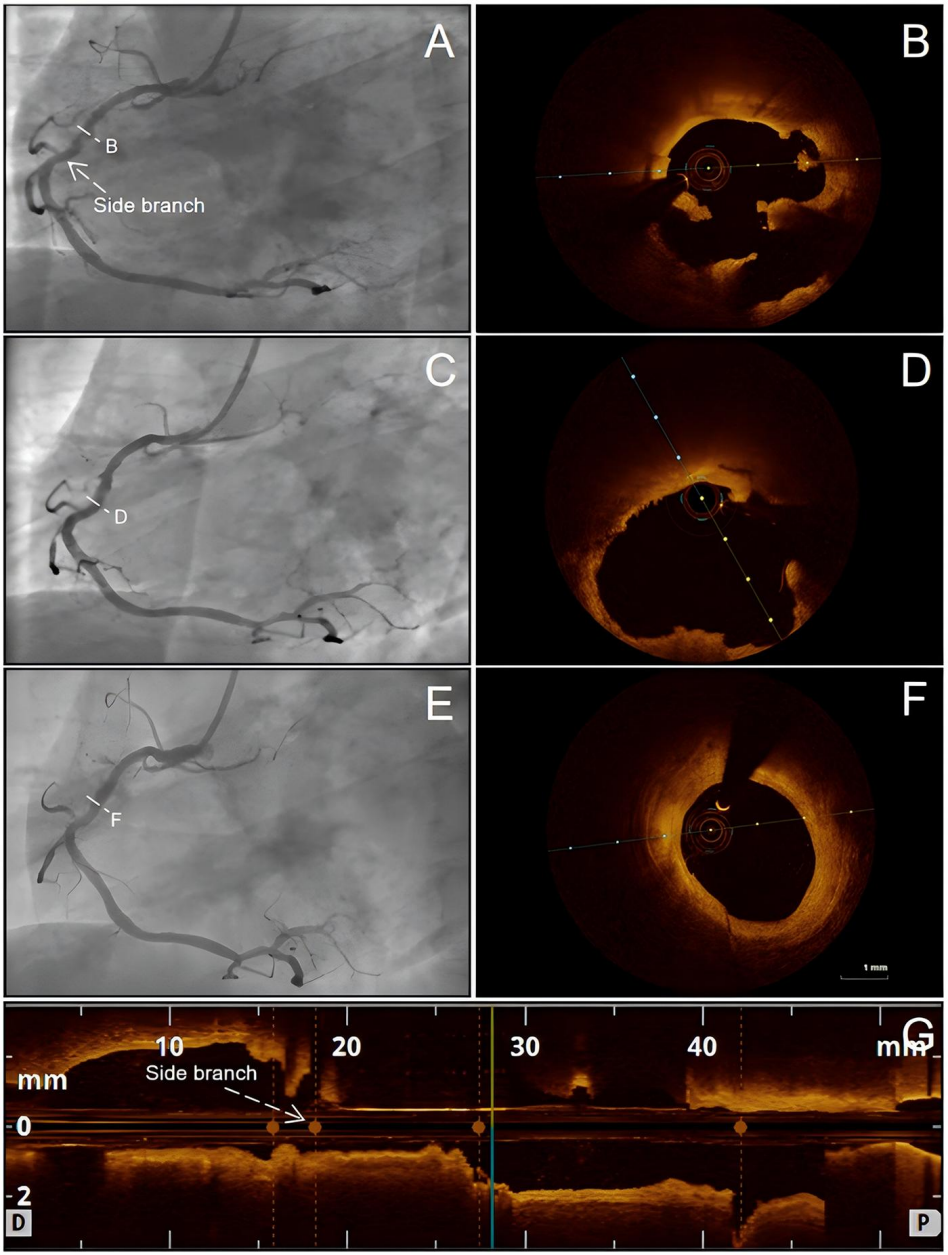
Serial OCT and CAG findings of the RCA at baseline, 1-month follow-up, and 1-year follow-up. At baseline, CAG of the RCA (after TA) showed moderate residual stenosis, and OCT revealed a ruptured plaque **(A,B)**. At 1-month follow-up, CAG showed moderate stenosis and OCT showed thrombus resorption **(C,D)**. At 1-year follow-up, CAG again demonstrated moderate stenosis, and OCT confirmed plaque stabilization with a thick fibrous cap and reduced lipid burden **(E,F)**. A longitudinal OCT image is provided to indicate the matched RCA segment/landmarks for serial comparison **(G)**. CAG, coronary angiography; OCT, optical coherence tomography; RCA, right coronary artery; TA, intracoronary thrombus aspiration.

The patient reported no significant discomfort and demonstrated good adherence to prescribed medications during the first month. At the 1-month follow-up, repeat CAG and OCT were performed. Repeat CAG showed persistent moderate residual narrowing of the proximal RCA (Figure 2C). The mild mid-LAD lesion remained angiographically stable, with no interval progression. OCT demonstrated an MLA of 4.52 mm^2^. Notably, at the previously identified plaque rupture site, thrombus resorption was observed; however, the minimal fibrous cap thickness remained thin, measuring 0.05 mm (Figure 2D, Supplementary Figure S1, Supplementary Video S2). In addition, the patient's LDL-C level decreased from 4.13 mmol/L at baseline to 2.54 mmol/L on day 15 after discharge and further to 1.80 mmol/L by day 30. Based on the repeat OCT and CAG findings, together with the patient's laboratory results and the absence of recurrent symptoms, stent implantation was again deferred.

**Figure 3:**
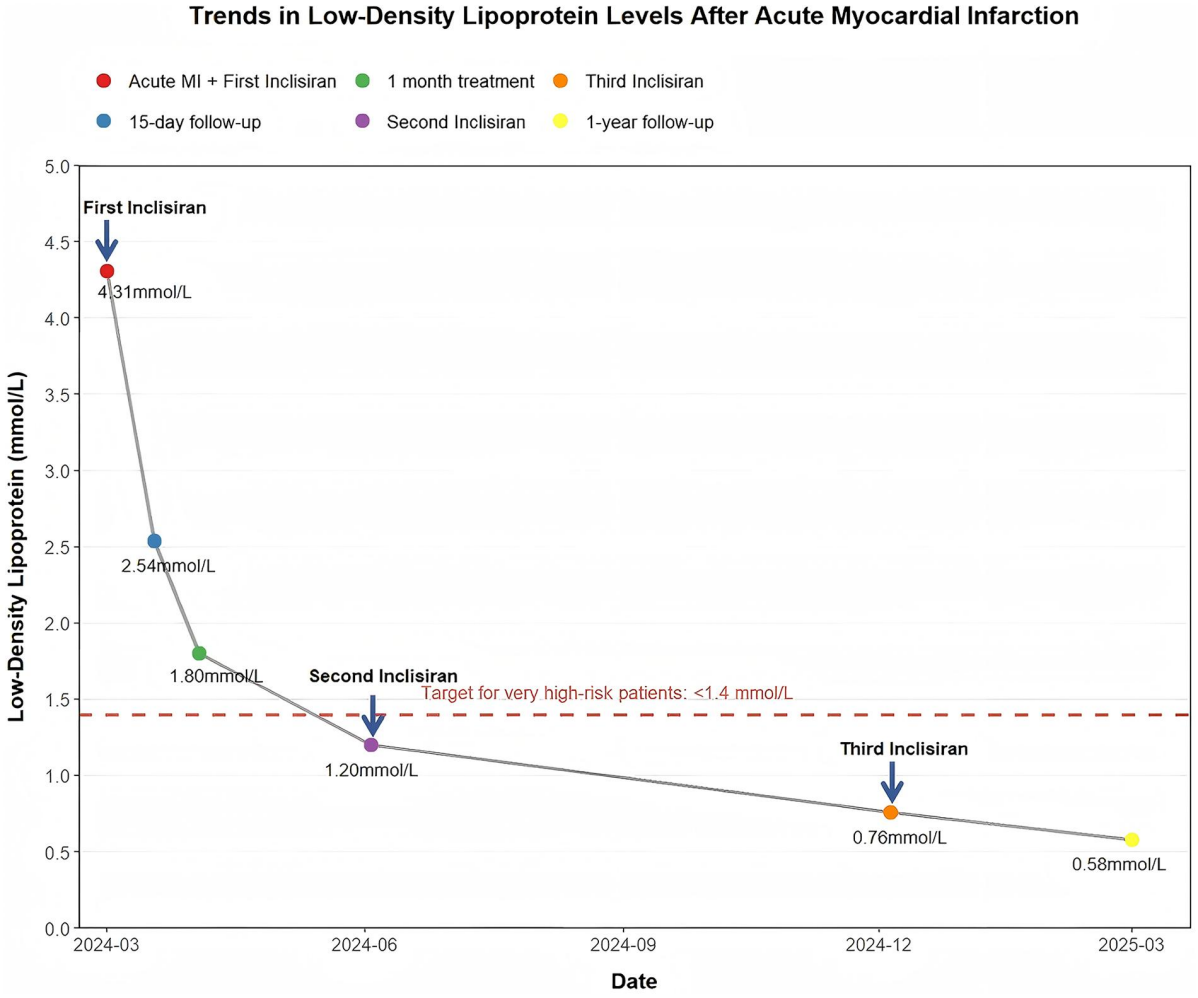
Line chart of the patient's serum lipid profile changes over a 1-year period. The chart illustrates the changes in the patient's LDL-C levels over 1 year and the three injections of inclisiran. LDL-C, low-density lipoprotein cholesterol.

At 3 and 9 months after discharge, the patient received the second and third subcutaneous injections of inclisiran (284 mg). LDL-C level decreased to 1.20 mmol/L at 3 months and remained stable thereafter (0.76 mmol/L at 9 months and 0.58 mmol/L at 1 year) (Figure 3). At the 1-year follow-up, CAG and OCT were performed to reassess coronary anatomy and plaque morphology and to guide further management. CAG demonstrated a moderate lesion in the proximal RCA (Figure 2E), broadly comparable to the angiographic appearance at the 1-month follow-up (Figure 2C). No progression was observed in the left coronary system. OCT demonstrated an MLA of 4.70 mm^2^. More notably, OCT revealed regression of the plaque at the culprit site into a stable phenotype, with a thicker fibrous cap and reduced lipid burden, compared with its appearance at the 1-month follow-up, when the initially ruptured plaque had healed but remained vulnerable, characterized by a thin fibrous cap and large lipid core. The minimal fibrous cap thickness increased from 0.05 mm at 1 month to 0.19 mm at 1 year (Figure 2F, Supplementary Figure S1, Supplementary Video S3). Given the absence of adverse events during the 1-year follow-up and OCT evidence of plaque stabilization, conservative management with guideline-directed medical therapy was continued. Throughout the 1-year follow-up, the patient remained clinically stable without recurrent chest pain or other ischemic symptoms. The clinical timeline is summarized in Table 1.

**Table 1:** Timeline.

Time point	Clinical events/management
At presentation	• The patient reported that he had developed chest pain 3 h prior.
15 min later	• Diagnosed with ST-segment elevation myocardial infarction. • Emergency CAG was performed followed by thrombus aspiration and OCT evaluation; stent implantation was deferred.
1 day later	• Intensive lipid-lowering therapy with inclisiran and atorvastatin was administered.
7 days later	• The patient had no obvious discomfort and was discharged from the hospital.
1 month later	• The patient underwent follow-up CAG and OCT, which revealed that the thrombus had been resorbed. The patient's LDL-C level was reduced to 1.8 mmol/L.
3 months later	• The second dose of inclisiran was administered to the patient. • The patient's LDL-C level was reduced to 1.2 mmol/L.
9 months later	• The third dose of inclisiran was administered to the patient. • The patient's LDL-C level was reduced to 0.76 mmol/L.
1 year later	• The patient was readmitted for a follow-up examination and the OCT revealed that the vulnerable plaque underwent reversal to a stable plaque phenotype. • The patient's LDL-C level was reduced to 0.58 mmol/L, with no discomfort reported.

## 3 Discussion

In this report, we describe a patient with acute inferior STEMI in whom serial OCT documented plaque healing and stabilization under intensive lipid-lowering therapy. After restoration of coronary flow by thrombus aspiration, OCT identified rupture of a vulnerable plaque as the underlying mechanism of the acute coronary event. Under intensive lipid-lowering therapy combined with dual antiplatelet treatment, serial OCT examinations documented a stepwise morphological transition of the culprit lesion—from an acutely ruptured plaque to a healed but still vulnerable phenotype at early follow-up, and ultimately to a stable plaque with fibrous cap thickening at 1 year. This case illustrates that sustained intensive lipid-lowering therapy was associated with plaque healing and stabilization, underscoring its role as a key component of contemporary ACS management. Serial OCT can visualize these dynamic morphological changes and support individualized clinical decision-making in selected patients.

Vulnerable plaques constitute a key underlying pathological substrate of ACS, and their identification and management have important implications for risk stratification and clinical decision-making (8). Vulnerable plaques are characterized by a thin fibrous cap (<65 μm), a large lipid necrotic core (>40% of plaque area), and prominent inflammatory infiltration. A thin fibrous cap is particularly susceptible to rupture under shear stress, and exposure of the lipid-rich core promotes platelet aggregation, precipitating abrupt coronary occlusion and the clinical presentation of ACS (9). Plaque rupture is an acute pathological event and accounts for nearly 70% of acute coronary events (2). The PROSPECT II study demonstrated that, even among non-culprit lesions in patients with ACS who had undergone successful treatment of the culprit lesion, features such as high plaque burden (≥70%), large lipid cores (lipid core burden index ≥324.7), thin fibrous caps (<65 μm), and positive remodeling were strong predictors of future cardiovascular events (10). Beyond the culprit lesion, future clinically relevant events may arise from non-culprit disease elsewhere in the coronary tree. The lipid-rich plaque (LRP) study showed that lipid-rich non-culprit plaques were associated with subsequent non-culprit lesion-related events, supporting the prognostic relevance of remote lesions beyond the culprit site (11). Building on the LRP experience, a focused report further highlighted that lipid-rich non-culprit segments—particularly within the LAD—were associated with a higher risk of subsequent non-culprit lesion-related events (12). Nevertheless, there remains no consensus on whether prophylactic stent implantation should be performed for vulnerable plaques without prior clinical events and with negative fractional flow reserve. The PREVENT trial reported that prophylactic stent implantation combined with optimal medical therapy reduced the risk of clinical endpoints, including repeat revascularization and hospitalization for angina, compared with optimal medical therapy alone (13). In our patient, the only additional finding was a mild, non-obstructive mid-LAD lesion, which remained angiographically stable during serial follow-up. In the present case, the initial OCT examination revealed a ruptured fibrous cap, a large false lumen, and extensive white thrombus at the culprit site, indicating that the patient's ACS resulted from rupture of an underlying plaque exhibiting vulnerable morphological features, leading to acute coronary occlusion.

LLT is the cornerstone of vulnerable plaque management in patients with ACS. Lower achieved LDL-C levels can increase fibrous cap thickness (particularly in plaques with thin caps at baseline), reduce macrophage infiltration, decrease lipid burden, and promote the transition of thin-cap fibroatheromas toward a more stable plaque phenotype, thereby reducing the incidence of major adverse cardiovascular events (MACE) in patients with ACS (5, 14, 15). A meta-analysis demonstrated that LLT significantly reduced percent atheroma volume, with the greatest reduction (1.56%) observed when follow-up LDL-C levels were ≤55 mg/dL. LLT also increased fibrous cap thickness, particularly when LDL-C was <70 mg/dL, with a mean increase of 66.9 μm (16). Taken together, these findings support intensive LDL-C lowering to achieve levels <55 mg/dL in very-high-risk patients, where imaging studies have shown greater plaque regression and stabilization (16). In addition, plaque regression and stabilization during LDL-C lowering can also be captured by coronary computed tomography angiography, although spatial resolution and plaque component characterization differ across imaging modalities (17). However, real-world studies have shown that only 28%–56% of high-risk patients with atherosclerotic cardiovascular disease (ASCVD) achieve recommended LDL-C targets, underscoring a persistent gap between guideline recommendations and routine lipid control (4).

Although statins are the cornerstone of LLT and can substantially reduce LDL-C levels, doubling the statin dose only increases the lipid-lowering effect by approximately 6%. This limited dose–response relationship is particularly relevant in high-risk patients. Moreover, concerns regarding adverse effects, including hepatic dysfunction and muscle-related symptoms, may limit further dose escalation in clinical practice. The application of PCSK9 monoclonal antibodies has achieved significant progress in intensive LLT. However, their long-term adherence and long-term immunological safety have restricted their application to a certain extent. With accumulating clinical experience, lipid variability during PCSK9 monoclonal antibody therapy has drawn increasing attention, as such fluctuations may influence therapeutic efficacy (18, 19). Inclisiran offers an alternative lipid-lowering approach with a distinct mechanism of action. As the first small interfering RNA therapeutic targeting PCSK9, inclisiran suppresses hepatic PCSK9 synthesis, thereby enhancing LDL receptor-mediated clearance of LDL-C (20). Data from randomized trials and real-world studies have shown that inclisiran produces substantial and sustained LDL-C reduction, with favorable adherence profiles and relatively stable lipid-lowering effects across dosing intervals (21, 22). However, whether inclisiran translates LDL-C lowering into long-term reductions in MACE among patients with ASCVD remains uncertain. In the present case, combination therapy with inclisiran and atorvastatin was associated with rapid attainment of LDL-C targets, reaching 1.2 mmol/L at 3 months, with sustained lipid control over 1 year.

The high spatial resolution of OCT enables direct measurement of fibrous cap thickness and identification of lipid-rich cores (low-signal areas), which are key morphological indicators of plaque vulnerability (3). Moreover, the excellent reproducibility of OCT satisfies the methodological requirements for serial follow-up, allowing precise and dynamic assessment of changes in plaque composition over time (23). In this case, IVUS was not performed. Nevertheless, intravascular ultrasound (IVUS) could have provided information complementary to OCT, particularly with respect to quantitative assessment of overall plaque burden, evaluation of vessel remodeling, and visualization of deeper arterial wall architecture using external elastic membrane-based measurements. Such insights are especially informative for diffuse atherosclerotic involvement and global vessel features (24). By contrast, OCT was selected after thrombus aspiration because the immediate goal at the index procedure was high-resolution intravascular characterization of the culprit lesion beyond angiography, allowing confirmation of plaque rupture and detailed assessment of the plaque–thrombus interface, including residual intraluminal thrombus. Building on this initial assessment, serial OCT across three time points documented a progressive evolution of plaque features—from rupture to a healed but still vulnerable phenotype, and ultimately toward a more stable morphology. This highlights the value of longitudinal OCT in dynamically assessing plaque healing and supporting continued GDMT.

## 4 Conclusion

Vulnerable plaques play a central role in the development of acute coronary events, emphasizing the importance of accurate identification and appropriate management. Sustained and effective LLT is closely linked to plaque stabilization and favorable remodeling, with lower achieved LDL-C levels correlating with more pronounced regression. Given its high spatial resolution and excellent reproducibility, serial OCT provides a valuable means for longitudinal assessment of plaque composition and stability, and may help inform individualized management strategies in patients with acute coronary syndromes.

## Data Availability

The original contributions presented in the study are included in the article/Supplementary Material, further inquiries can be directed to the corresponding authors.

## Supplementary material

Supplementary Figure S1 Minimal fibrous cap thickness at the same site in the RCA as measured by OCT at 1-month and 1-year follow-up. Panel **(A)** shows the minimal fibrous cap thickness of the RCA as assessed by OCT at the 1-month follow-up, measuring 0.05 mm (left panel). Panel **(B)** shows the minimal fibrous cap thickness of the RCA as assessed by OCT at the 1-year follow-up, measuring 0.19 mm (right panel). OCT, optical coherence tomography; RCA, right coronary artery.

Supplementary Video S1 Baseline OCT.

Supplementary Video S2 One-month follow-up OCT.

Supplementary Video S3 One-year follow-up OCT.
