# Supplementary figures and images for: Intensive lipid-lowering therapy-related regression of a vulnerable plaque confirmed by serial optical coherence tomography: a case report
Source: Front Cardiovasc Med. 2026 Feb 26;13:1696537. doi: 10.3389/fcvm.2026.1696537 (PMC12979529; doi:10.3389/fcvm.2026.1696537)

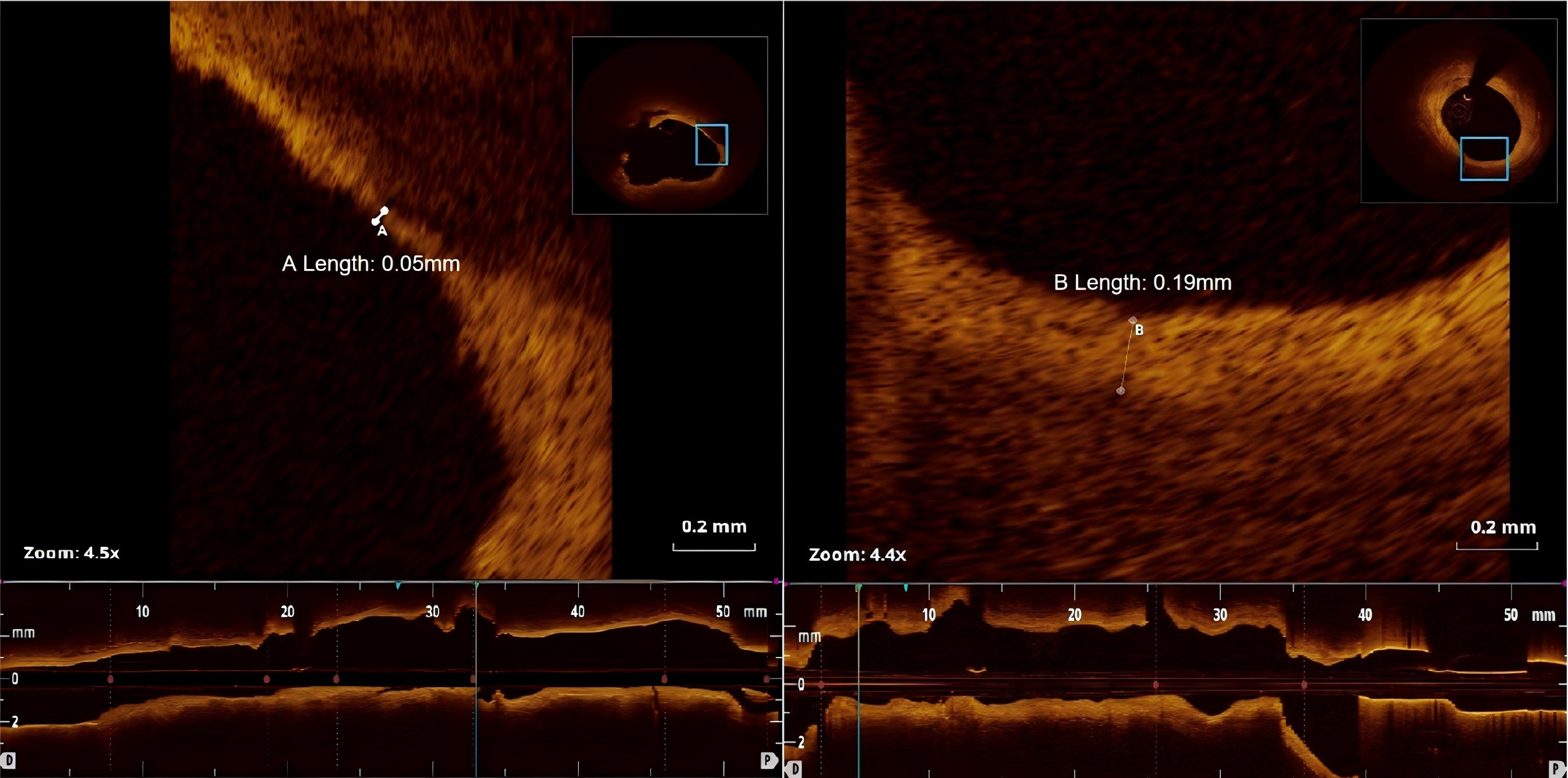

Supplement: Supplementary Figure S1 — Minimal fibrous cap thickness at the same site in the RCA as measured by OCT at 1-month and 1-year follow-up. Panel (A) shows the minimal fibrous cap thickness of the RCA as assessed by OCT at the 1-month follow-up, measuring 0.05 mm (left panel). Panel (B) shows the minimal fibrous cap thickness of the RCA as assessed by OCT at the 1-year follow-up, measuring 0.19 mm (right panel). OCT, optical coherence tomography; RCA, right coronary artery. [file Image1.jpeg]
